# Hydroxychloroquine Attenuates Myocardial Ischemic and Post-Ischemic Reperfusion Injury by Inhibiting the Toll-Like Receptor 9 – Type I Interferon Pathway

**DOI:** 10.26502/fccm.92920278

**Published:** 2022-08-25

**Authors:** Katherine M Marsh, Radhika Rastogi, Aimee Zhang, Di Wu, Irving L Kron, Zequan Yang

**Affiliations:** 1 Department of Surgery, University of Virginia, Charlottesville, VA, USA

**Keywords:** Hydroxychloroquine, Ischemia Reperfusion Injury, Myocardial Infarction, Toll-Like Receptor 9, Type I Interferon

## Abstract

**Background:**

We hypothesized that hydroxychloroquine (HCQ) attenuates myocardial ischemia/reperfusion injury (IRI) via TLR9 – type I interferon (IFN-I) pathway inhibition.

**Methods:**

The left coronary artery of wild-type (WT) C57BL/6 and congenic TLR9^−/−^ mice was occluded for 40 minutes, with or without 60 minutes of reperfusion (40’/0’ or 40’/60’). Either ODN-2088 or HCQ (TLR9 inhibitors), or ODN-1826 (TLR9 agonist) was administered to determine effect on infarct size (IS). After 40’/0’, cardiac perfusate (CP) was collected from harvested hearts and administered to either intact WT mice after 20 minutes of ischemia or isolated splenocytes. Type-I interferon (IFNα and IFNβ) levels were measured in plasma and splenocyte culture supernatant, and levels of damage associated molecular patterns HMGB1 and cell-free DNA (cfDNA) were measured in CP.

**Results:**

After 40’/60’, WT mice treated with HCQ or ODN-2088 had significantly reduced IS. TLR9^−/−^ mice and HCQ-treated WT mice undergoing 40’/0’ and 40’/60’ similarly attenuated IS, with significantly lower IFN-Is in CP after 40’/0’ and in plasma after 40’/60’. IS was significantly increased in 40’/0’ CP-treated and ODN-1826-treated 20’/60’ WT mice. CP-treated WT splenocytes produced significantly higher IFN-I in culture supernatant, which was significantly reduced with HCQ.

**Conclusions:**

The TLR9–IFN-I-mediated inflammatory response contributes significantly to both ischemic and post-ischemic myocardial ischemia-reperfusion injury. HMGB1 and cfDNA released from ischemic myocardium activated the intra-myocardial TLR9 – IFN-I inflammatory pathway during ischemia and the extra-myocardial TLR9 – IFN-I inflammatory pathway during reperfusion. Hydroxychloroquine reduces production of IFN-I and attenuates myocardial IRI, likely by inhibiting the TLR9–IFN-I pathway.

## 1. Introduction

Immune-mediated myocardial injury has been implicated in a broad range of cardiac diseases including ischemic heart disease [1–4]. Mounting clinical evidence suggests that acute myocardial infarction (MI), myocardial ischemia/reperfusion injury (IRI), and post-MI left ventricular (LV) remodeling are all associated with enhanced immune responses [4–9]. Activation of the innate immune-mediated inflammatory response occurs when damage-associated molecular patterns (DAMPs) released from the damaged cardiomyocytes interact with pattern recognition receptors (PRRs). Among PRRs, Toll-Like Receptors (TLR) in particular have been recognized as key elements to mediate the inflammatory response in acute MI and myocardial IRI [3, 10]. Once released into the bloodstream during post-ischemic reperfusion, DAMPs such as cell-free DNA (cfDNA) and high mobility group box-1 (HMGB1) activate the receptor for advanced glycation endproducts (RAGE) – TLR9 pathway to invoke an inflammatory response [11, 12]. cfDNA additionally activates plasmacytoid dendritic cells (pDCs) to release type I interferons (IFN-Is) including interferon alpha and beta (IFNα and IFNβ). In turn, IFN-Is mediate post-ischemic reperfusion injury [13].

The anti-malarial drug hydroxychloroquine (HCQ) is a TLR7 and TLR9 antagonist [14] that has been used as an immunomodulator to treat autoimmune diseases such as systemic lupus erythematosus [15–17] and rheumatoid arthritis [18]. HCQ exhibits potent anti-inflammatory properties by inhibiting TLR9 and IFN-Is within pDCs [17]. HCQ has also demonstrated protection against renal [19], skeletal muscle [20], and myocardial [21] IRI when administered before onset of ischemia, but the role of HCQ after the onset of myocardial infarction remains unknown. During post-ischemic reperfusion injury, the pDC-IFN-I pathway has been shown to mediate the inflammatory response [13]. Thus, it is worthwhile to evaluate the role of HCQ in protecting the heart against both acute MI and subsequent reperfusion injury.

We hypothesized that the TLR9 – IFN-I pathway is activated during acute MI and mediates both ischemic myocardial injury and post-ischemic reperfusion injury. Since HCQ inhibits the TLR9 – IFN-I pathway, we further hypothesize that administration of HCQ will attenuate the inflammatory response and associated post-ischemia reperfusion injury. If administered before the onset of ischemia, it should additionally attenuate ischemic injury from MI.

## 2. Materials and Methods

This study complied with the 2011 Guide for the Care and Use of Laboratory Animals, 8th edition as recommended by the U.S. National Institutes of Health ensuring that all animals received humane care. The University of Virginia Animal Care and Use Committee reviewed and approved the study protocol (#3943-08-18).

### 2.1 Animals and Materials

C57BL/6 wild type (WT) mice and TLR9^−/−^ mice (male, aged 9–14 weeks, purchased from The Jackson Laboratory, Bar Harbor, ME) were used in the study. HCQ was purchased from ThermoFisher (Waltham, MA). Oligodeoxynucleotides (ODN)-1826, ODN-2088, and ODN-2088 negative control were purchased from InvivoGen (San Diego, CA). Anti-HMGB1 antibodies were purchased from Abcam (Cambridge, UK). IFNα and IFNβ antibodies were purchased from ThermoFisher (Waltham, MA).

### 2.2 Experimental Groups

#### 2.2.1 Ischemia-only (40’/0’):

**Figure 1:**
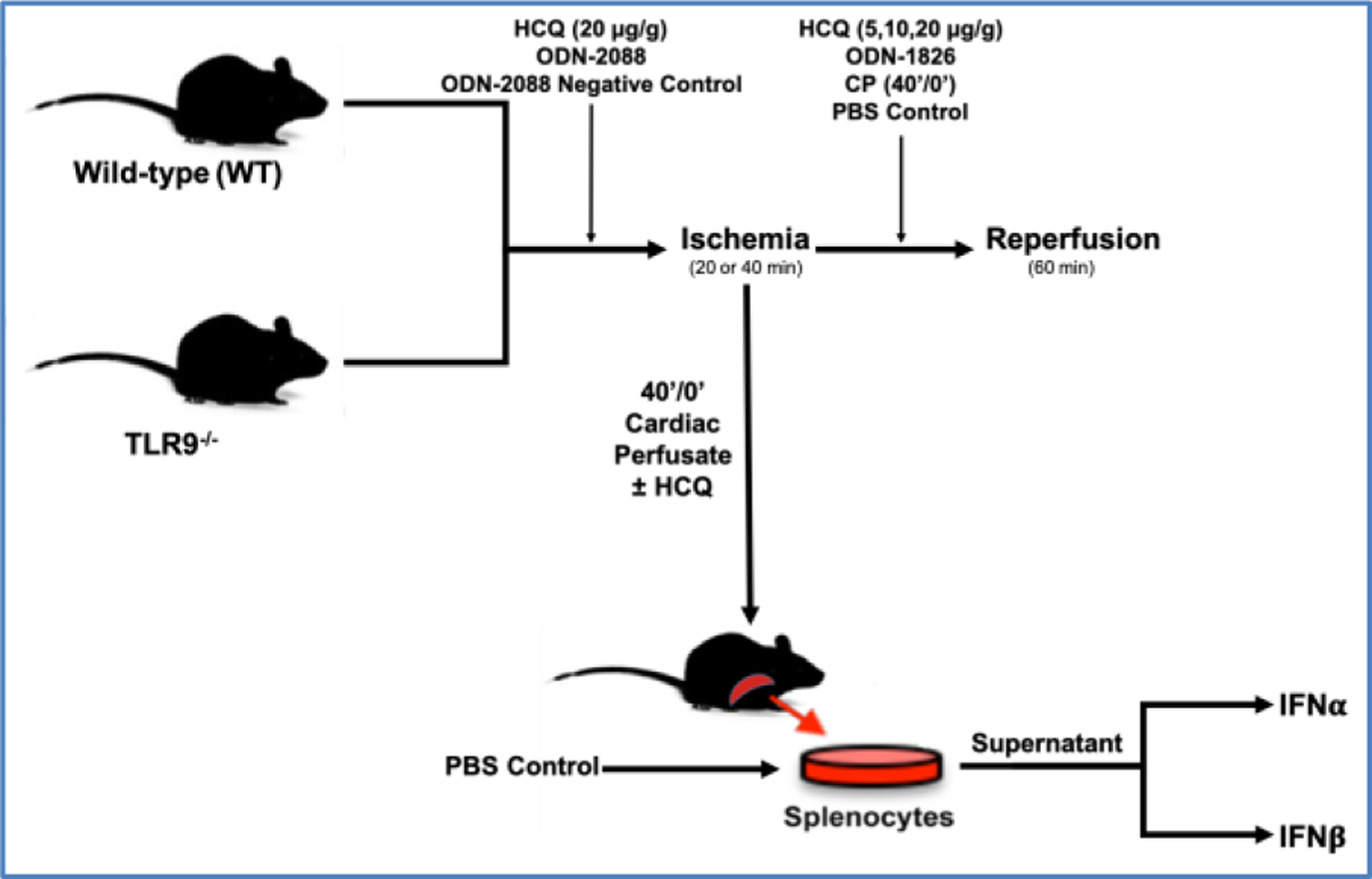
Experimental protocol. C57BL/6 (WT) and TLR9^−/−^ mice underwent 20 or 40 min of ischemia by left coronary artery occlusion with or without 60 min of reperfusion before infarct size was evaluated by TTC-Phthalo blue staining. Treatment groups were treated with hydroxychloroquine (HCQ), ODN-2088 (TLR antagonist), or ODN-2088 negative control prior to ischemia or HCQ, ODN-1826 (TLR 9 agonist), or control prior to reperfusion. After 40’/0’, CP was harvested and then used to treat WT mice or splenocytes with or without HCQ, after which type-I interferon (IFNα and IFNβ) levels were measured.

WT and TLR9^−/−^ mice were treated with either phosphate buffered saline (PBS) (2 μl/g) or high dose HCQ (20 μg/g) 5 minutes prior to ischemia. The mice then underwent 40 minutes of ischemia without reperfusion (Figure 1). Cardiac perfusate (CP) was collected from these mice before TTC-Blue staining, discussed further below.

#### 2.2.2 IRI Pre-Ischemic Treatment (40’/60’):

WT mice were pre-treated 5 minutes prior to left coronary artery (LCA) occlusion with either high dose HCQ (20 μg/g), ODN-2088 negative control (0.6 μg/g), or ODN-2088 (0.6 μg/g). Mice then underwent 40 minutes of ischemia and 60 minutes of reperfusion (Figure 1).

#### 2.2.3 IRI Pre-Reperfusion Treatment (40’/60’ or 20’/60’):

WT mice underwent 40 minutes of ischemia, followed by treatment with either 2μl/g body weight PBS (control), or varied dosages of HCQ (5 μg/g, 10 μg/g, or 20 μg/g body weight) as an intravenous bolus 5 minutes before reperfusion. Lastly, the mice underwent 60 minutes of reperfusion (Figure 1). A separate group of WT mice underwent 20 minutes of ischemia and then treatment 5 minutes before reperfusion with either PBS (control, 2 μl/g), 40’/0’ CP (2 μl/g), or ODN-1826 (10 μg/g). The mice then underwent 60 minutes of reperfusion (Figure 1).

#### 2.2.4 Ex-Vivo Experiment:

CP was collected in WT mice that underwent 40 minutes of LCA occlusion without reperfusion (40’/0’). Isolated WT splenocytes were treated either with PBS, CP, or CP with HCQ, discussed further below. After 2 hours, IFNα and IFNβ levels were measured in the cell culture supernatant (Figure 1).

### 2.3 Myocardial IRI

Myocardial infarction was induced in intact mice as previously described[4, 22, 23]. Briefly, anesthetized mice (Avertin 250 mg/kg with an additional 125mg/kg dose every 30 minutes intraperitoneally) were placed in a supine position on a heating pad, orally intubated with a PE-60 tube, and mechanically ventilated at a tidal volume of 10 μl/g and rate of 130 stroke/minute (MiniVent Ventilator, Harvard Apparatus, Holliston, MA). A left thoracotomy was performed by cutting the left 3^rd^ and 4^th^ ribs and intercostal muscles to expose the heart. An 8–0 Prolene suture was passed underneath the LCA at the level of the lower edge of the left atrium and tied over a piece of PE-50 tubing to occlude the LCA for 20 or 40 minutes. Successful LCA occlusion was confirmed by color change in the region at risk. Reperfusion was achieved by removing the tubing. A volume of 1–1.5 ml 5% dextrose was given intraperitoneally to replace insensible losses during the operation. Core body temperature was monitored throughout the operation with a rectal thermocouple interfaced to a digital thermometer (Barnant Co, Barrington, IL) and maintained between 36.5–37.5°C.

### 2.4 Determination of Infarct Size

Following LCA occlusion with or without reperfusion, mice were euthanized under deep anesthesia. The heart was isolated and ascending aorta cannulated with a blunt 23-gauge needle. The heart was sequentially perfused with 3 ml of PBS (pH=7.4) and 3 ml 1% 2,3,5-Triphenyltetrazolium chloride (TTC) in PBS at 37°C. The LCA was then re-occluded by retying the LAD-encircling suture, and the heart was perfused with 0.5–1.0 ml 10% Phthalo Blue (Heubach Ltd, Fairless Hills, PA) to delineate the non-ischemic region. The heart was then frozen and trimmed of the right ventricle and atria. The left ventricle was cut into 5–7 slices, which were fixed in 10% neutral buffered formalin solution. Each slice was weighed and photographed. The sizes of the non-ischemic area, the area at risk, and the infarct area were calculated as a percentage of the corresponding area multiplied by the weight of the slice as previously reported [4, 22–26]. For example, myocardial infarct size (IS) was reported as a percentage of risk region (RR, % of LV).

### 2.5 Cardiac Perfusate (CP) and Splenocyte Cell Culture

#### 2.5.1 Acquisition of Cardiac Perfusate (CP):

Following 40 minutes of ischemia without reperfusion (40’/0’), the hearts were harvested, and their ascending aorta was cannulated with a 23-gauge needle. Hearts were then perfused with 500 μl PBS (pH=7.4 at 37°C) for 4 cycles. The CP was collected and centrifuged at 3000 rpm for 20 minutes to discard cellular sediments. CP levels of cfDNA and HMGB1 were evaluated using Nanodrop and Western Blot (Anti-HMGB1 antibody purchased from Abcam, Cambridge, UK) [11,12].

#### 2.5.2 Splenocyte Culture with Stimulation by CP:

The spleen was dissociated into a single-cell suspension using PBS supplemented with 10% fetal bovine serum in GentleMACs tubes (Miltenyi Biotec). Red blood cells were lysed by re-suspending splenic cells in ammonium chloride-Tris buffer and incubating them at room temperature for 8 minutes. Leukocytes were collected following centrifugation and washed twice in PBS. The splenic leukocytes were resuspended in PBS, enriched to 2 × 10^6^/μl and incubated in 6-well plates (BD Company) at a concentration of 8.2×10^6^ cells in 1.2 ml of culture media per well. Then, 100 μl of PBS or 40′/0′ CP (containing 8.1 μg/ml cfDNA), respectively, were added to 4‐well (n=4/group) cell culture plates and incubated for 2 hours. Another group of splenocytes were pretreated with HCQ (92 μM) administered 30 minutes before the addition of 40′/0′ CP and then incubated for 2 hours. Following the incubation period, supernatants were collected and the levels of IFNα and IFNβ were measured by Western blot. Live counts of splenic leukocytes were measured using a fluorescence automated cell counter (Cellometer K2, Nexcelom. Lawrence, MA).

### 2.6 Measurement of IFNα, IFNβ, HMGB1 and cfDNA

Plasma and CP levels of IFNα and IFNβ were determined using an ELISA kit (Bio-Rad Laboratories). Levels of IFNα and IFNβ in the supernatant of splenic leukocyte cultures and levels of HMGB1 in CP were evaluated using Western Blot (ThermoFisher). Levels of cfDNA in CP were measured using Nanodrop.

### 2.7 Statistical Analysis

Comparisons between groups were performed with one-way analysis of variance with Bonferroni’s correction for multiple comparisons and unpaired Student’s t-test. Prism 7 (GraphPad Software Inc., La Jolla, CA) was used to perform statistical calculations. Data are presented as mean±standard error of the mean, with a *p*-value < 0.05 indicating statistical significance.

## 3. Results

### 3.1 TLR9 and HCQ in Myocardial Ischemic Injury

**Figure 2:**
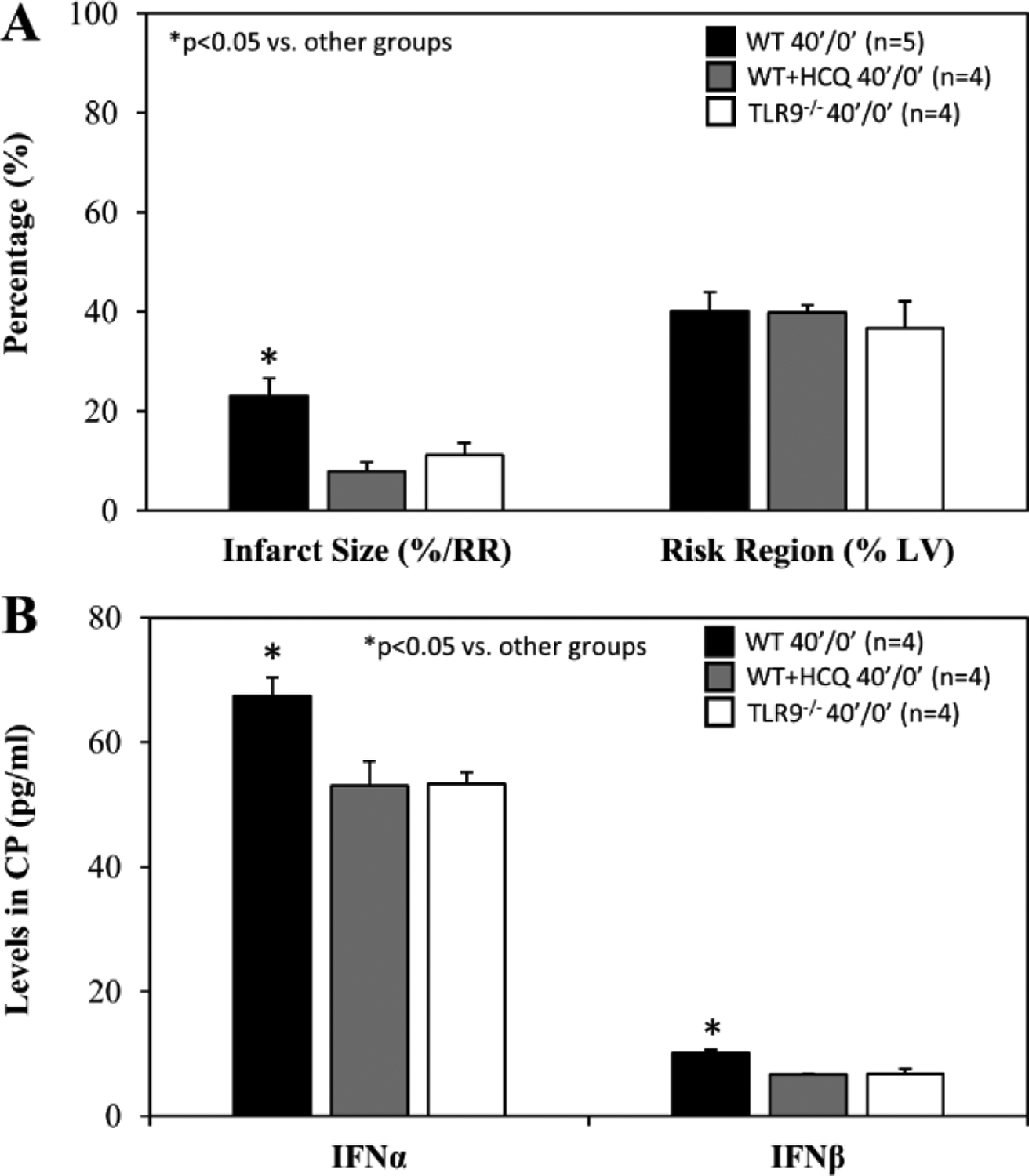
Infarct size in TLR9^−/−^ mice and after hydroxychloroquine treatment in WT mice after acute myocardial infarction. Wild type (WT) mice, WT mice treated with hydroxychloroquine (HCQ), and congenic TLR9^−/−^ mice underwent 40 min ischemia of the left coronary artery without reperfusion (40’/0’). **A**. Infarct size (IS), as a percent of risk region (RR), and RR, as a percent of left ventricle (LV), were measured with TTC-phthalo-blue staining, and **B.** levels of interferon α (IFNα) and interferon β (IFNβ) in the cardiac perfusate (CP) were measured for each group.

**Figure 3:**
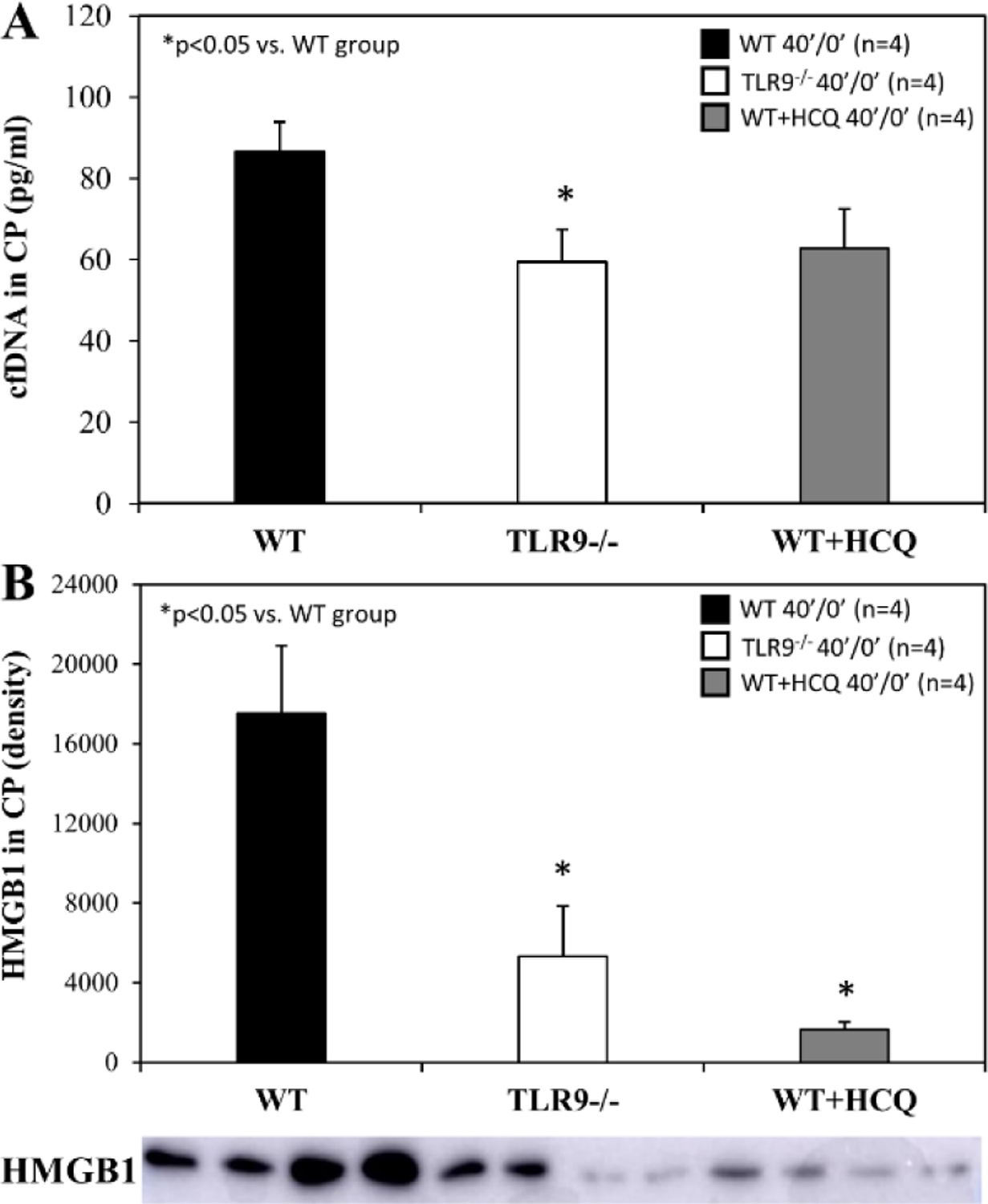
HMGB1 and cfDNA in TLR9^−/−^ mice and after hydroxychloroquine treatment in WT mice after myocardial infarction. Wild type (WT) mice, WT mice treated with hydroxychloroquine (HCQ), and congenic TLR9^−/−^ mice underwent 40 min ischemia of the left coronary artery without reperfusion (40’/0’). Cardiac perfusate was harvested from each group and levels of the damage associated molecular patterns **A**. cell-free DNA (cfDNA) and **B.** high mobility box 1 (HMGB1) were measured for each group.

For mice that underwent 40 minutes of ischemia without reperfusion (40’/0’), ischemic risk region (RR, as percentage of LV mass) was comparable among all groups. Infarct size (IS) was measured as percentage of RR. WT control mice had an IS of 23±4% whereas treatment with HCQ, as a 20 μg/g intravenous bolus administered before occlusion, attenuated IS to 8±2% (*p*<0.05 vs. control). In TLR9^−/−^ mice, IS was 11±2%, a 50% reduction from WT control (*p*<0.05, Figure 2A). Levels of IFNα and IFNβ in CP were significantly lower in HCQ-treated WT mice and TLR9^−/−^ mice than WT control mice (Figure 2B). Cell-free DNA and HMGB1 in CP were also significantly lower in HCQ-treated WT mice and TLR9^−/−^ mice than WT control mice (Figure 3).

### 3.2 TLR9 and HCQ in Post-Ischemic Reperfusion Injury

**Figure 4:**
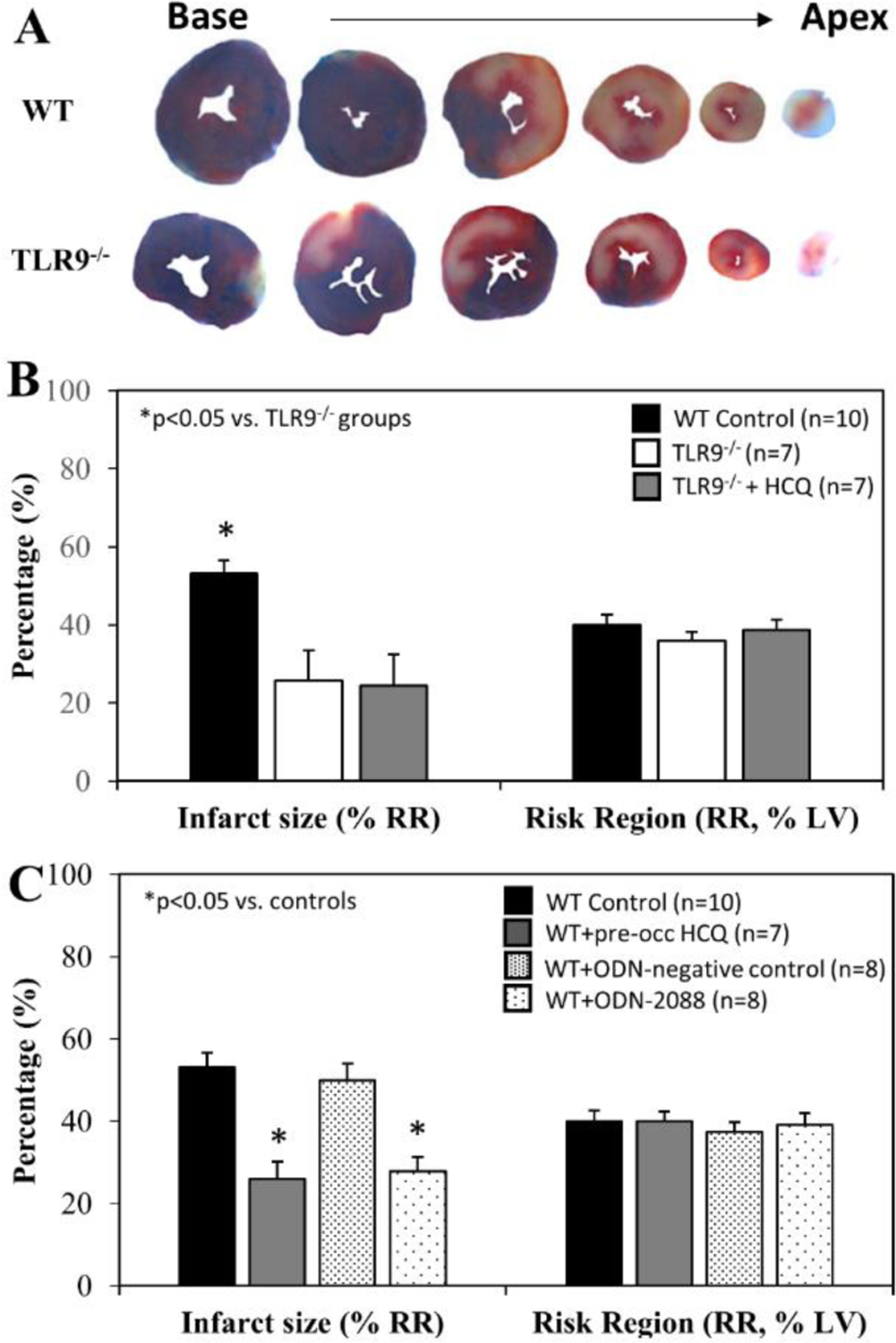
Infarct size in TLR9^−/−^ mice and WT mice with treatment. Wild type (WT) mice and congenic TLR9^−/−^ mice underwent 40 min ischemia of the left coronary artery with 60 min of reperfusion (40’/60’). **A.** Representative slices of the heart after myocardial IRI are shown for WT and TLR9^−/−^ mice. **B**. Infarct size (IS), as a percent of risk region (RR), and RR, as a percent of left ventricle (LV), were measured with TTC-phthalo-blue staining for WT mice, congenic TLR9^−/−^ mice and TLR9^−/−^ mice treated with hydroxychloroquine (HCQ). **C.** IS and RR were measured in WT controls, after treatment prior to ischemia with HCQ, after treatment prior to ischemia with ODN-2088 (TLR9 antagonist), and after treatment prior to ischemia with ODN-2088 negative controls.

When treated immediately prior to ischemia (IRI pre-ischemic treatment), IS after 60 minutes of reperfusion was significantly attenuated to 26±8% in TLR9^−/−^ mice, a >40% reduction compared to WT control mice (*p*<0.05). HCQ treatment (20 μg/g) did not further reduce the IS in TLR9^−/−^ mice (Figure 4A & 4B). Pre-treated WT mice with ODN-2088 or high dose HCQ similarly attenuated IS (28±4% or 26±2%, respectively, vs. corresponding control — ODN negative 50±4% or PBS control 53±4%, *p*<0.05. Figure 4C). RR was comparable among the control and treated groups (Figure 4C, right column).

**Figure 5:**
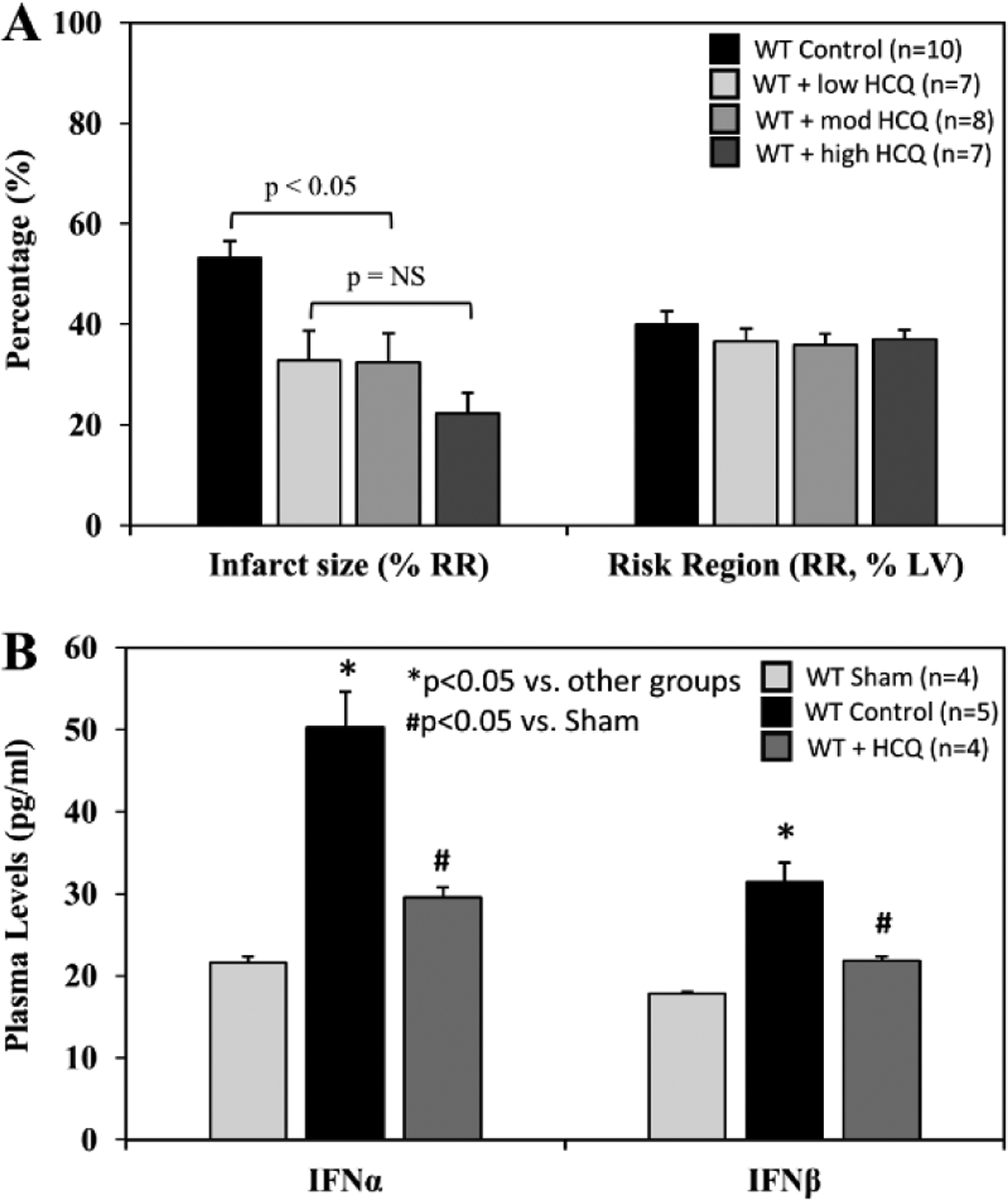
Infarct size and type-I interferon levels in WT mice with varying hydroxychloroquine treatment doses. Wild type (WT) mice underwent 40 min ischemia of the left coronary artery with 60 min of reperfusion (40’/60’). **A**. The mice were treated with hydroxychloroquine (HCQ) prior to reperfusion at low (5μg/g), moderate (10μg/g), and high (20μg/g) doses, and infarct size (IS), as a percent of risk region (RR), and RR, as a percent of left ventricle (LV), were measured with TTC-phthalo-blue staining. **B.** Levels of interferon α (IFNα) and interferon β (IFNβ) in the plasma for WT sham mice (thoracotomy without coronary occlusion), WT control mice, and WT mice after HCQ treatment prior to reperfusion were measured for each group.

When treated immediately prior to reperfusion (IRI pre-reperfusion treatment), RR was comparable among the control and 3 HCQ-treated groups (5 μg/g, 10 μg/g, or 20 μg/g) following 40 minutes of ischemia and 60 minutes of reperfusion (40’/60’). IS was significantly reduced in all HCQ-treated mice (low dose HCQ 33±3%, moderate dose 33±6%, and high dose 22±4%, vs. control 53±3%, *p*<0.05). High dose HCQ-treated mice tended to have a smaller IS compared to the low and moderate dose HCQ-treated mice, but the difference did not reach statistical significance (Figure 5A). In control mice, plasma levels of IFNα and IFNβ were significantly elevated at the end of 60 minutes of reperfusion. Levels of IFNα and IFNβ in HCQ-treated mice (moderate dose) were significantly lower than control mice but remained higher than the control mice (Figure 5B).

**Figure 6:**
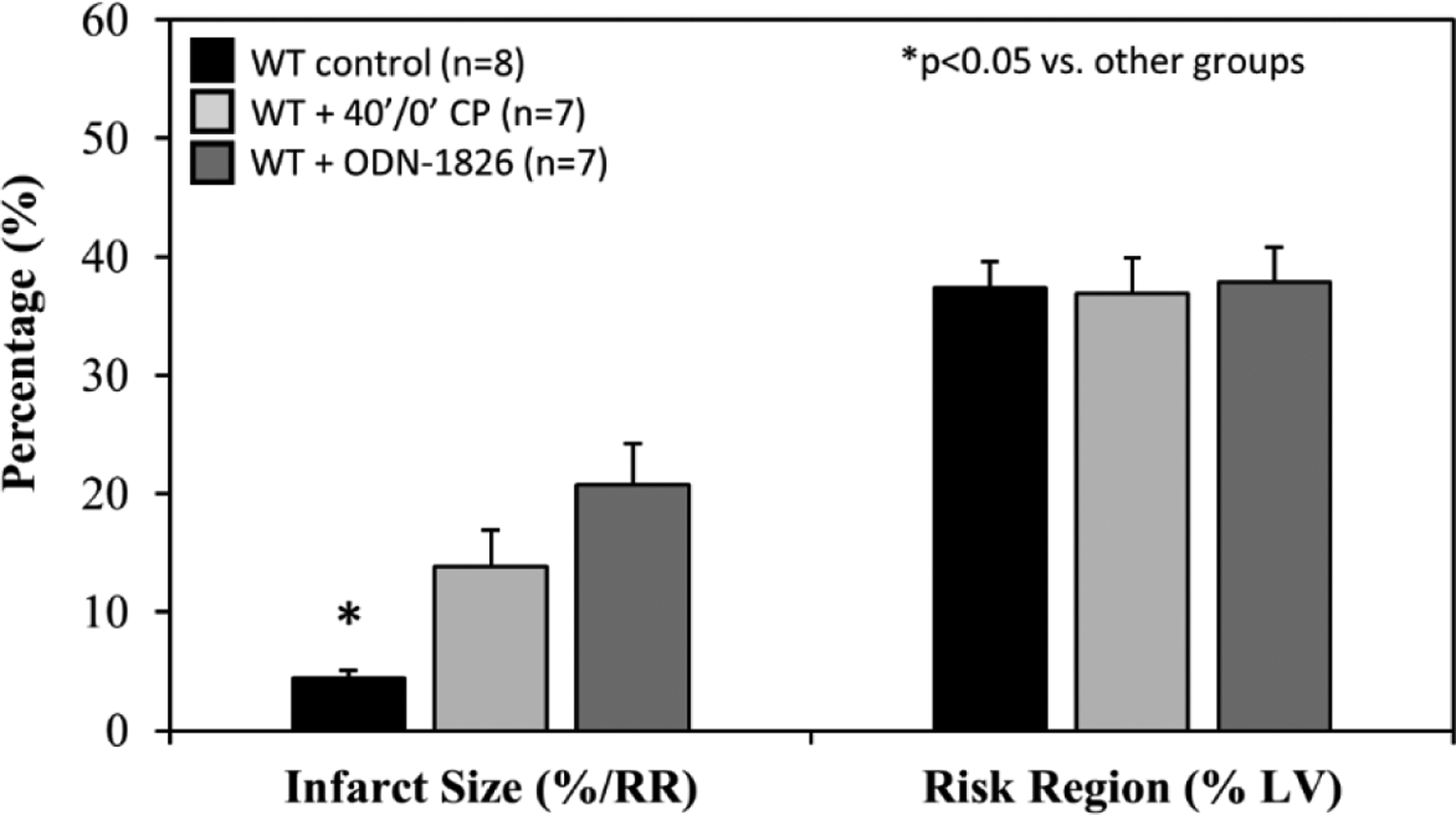
Infarct size after treatment with cardiac perfusate or TLR9 agonist ODN-1826. Wild type (WT) mice underwent 20 min ischemia of the left coronary artery with 60 min reperfusion (20’/60’). Prior to reperfusion, mice were treated with ODN-1826 (TLR agonist) or cardiac perfusate (CP) from 40’/0’ WT control mice. Infarct size (IS), as a percent of risk region (RR), and RR, as a percent of left ventricle (LV), were measured with TTC-phthalo-blue staining.

After 20 minutes of ischemia and 60 minutes of reperfusion (20’/60’) with IRI pre-reperfusion treatment, there were no difference in RR among all groups. The IS in WT control mice was 4±1%. CP and ODN-1826 significantly exacerbated IS to 14±3% and 21±4% respectively (*p*<0.05 vs. control). There was no statistical difference between CP- and ODN-1826-treated groups (Figure 6).

### 3.3 Cardiac Perfusate from Ischemic Hearts Stimulates Type I Interferon Responses

**Figure 7:**
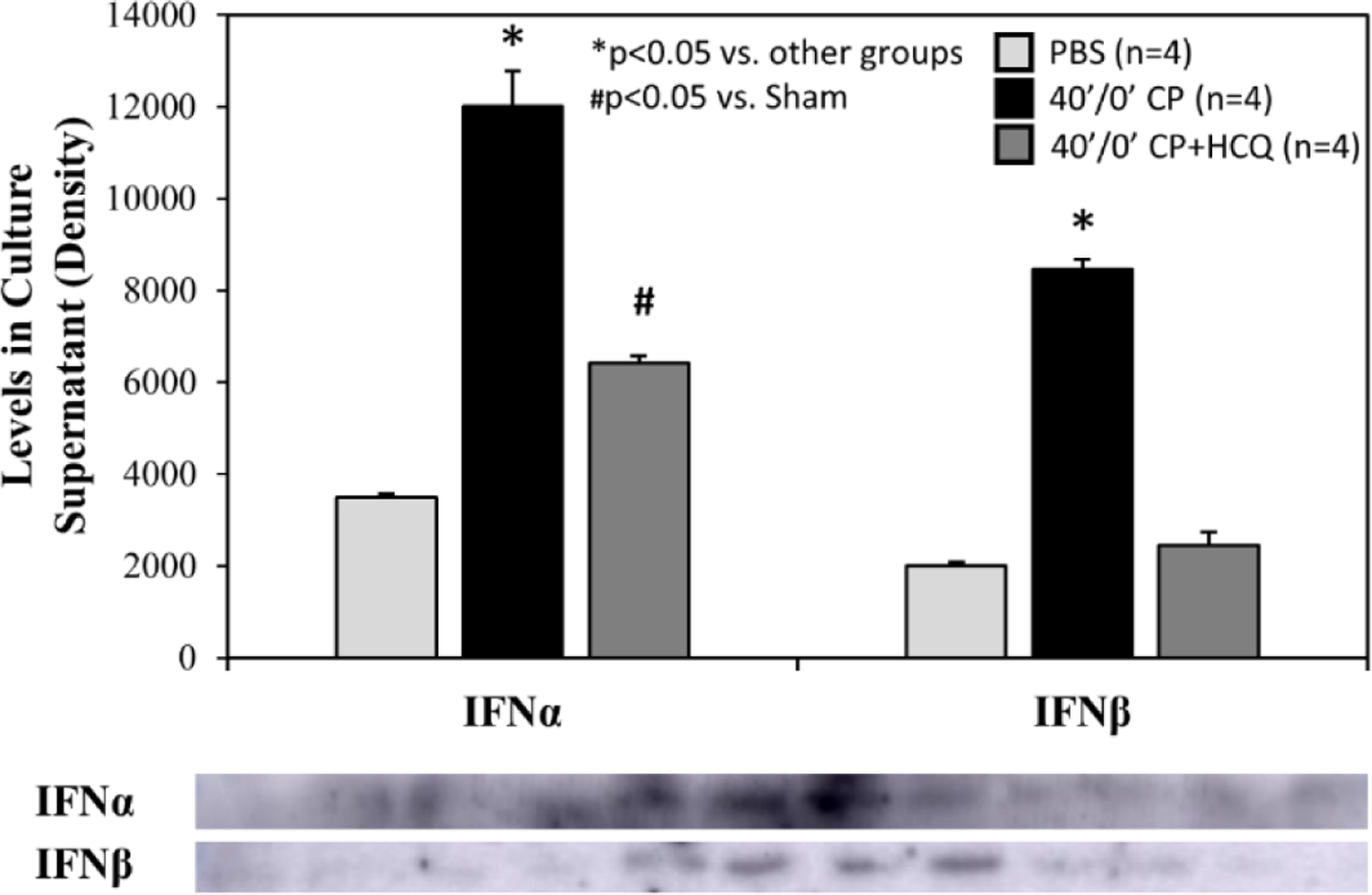
Levels of type-I interferons in splenocyte culture supernatant. Cardiac perfusate (CP) was harvested from wild type mice who underwent 40 min ischemia of the left coronary artery without reperfusion (40’/0’). Cultured splenocytes were then treated with PBS as a control, CP, or CP with hydroxychloroquine (HCQ) for 2 hours. Levels of interferon α (IFNα) and interferon β (IFNβ) in the splenocyte culture supernatant were then measured.

Splenic leukocytes were incubated in 6-well BD plates at a final concentration of 6.37×10^6^ cells in 1.3 ml of culture media plus treatment per well. After 2 hours of incubation, the splenocyte count dropped similarly in each group by an average of 13%. There were 5.56±0.08×10^6^ living splenocytes remaining with PBS control, 5.73±0.05×10^6^ with CP alone, and 5.34±0.17×10^6^ with CP plus HCQ (p=NS among the 3 different treatments). CP stimulated splenic leukocytes to secrete IFNα and IFNβ. The increases in IFNα and IFNβ levels were reduced significantly by treatment with HCQ (Figure 7).

## 4. Discussion

Our previous studies have demonstrated that ischemically-injured cardiomyocytes release DAMPs into the circulation which activate the pDC – IFN-I pathway, exacerbate the inflammatory response, and induce post-ischemic reperfusion injury. The present study further demonstrates that the inflammatory response is triggered by the TLR9 – IFN-I pathway both inside ischemic myocardium and systemically during post-ischemic reperfusion. HCQ exerts cardioprotective effects against both initial ischemic injury and subsequent post-ischemic reperfusion injury by inhibiting the TLR9 – IFN-I pathway.

Total myocardial infarction during ischemia and post-ischemic reperfusion is a function of ischemic infarction; i.e. there will be no cardiomyocytic necrosis during reperfusion if there is no initial ischemic myocardial necrosis [11, 27]. Myocardial ischemic injury has been investigated, to some extent, by isolated Langendorff heart models as they demonstrate *de novo* inflammatory responses inside the heart. Hypoxia-injured cardiomyocytes release DAMPs including mitochondrial DNA, which activate the TLR9 – IFN-I pathway and mediate the inflammatory response inside the myocardium [28]. *In vivo* myocardial IRI activates not only the intrinsic myocardial inflammatory response during ischemia, but also the extrinsic inflammatory responses during post-ischemic reperfusion, which collectively culminates in overall intramyocardial inflammation [4,11,22]. This helps explain why HCQ has demonstrated protection against myocardial IRI when administered before the onset of ischemia[21]. In the current study, we found that both IFNα and IFNβ levels were significantly elevated in 40’/0’ CP (Figure 2), 40’/60’ plasma (Figure 5) and 40’/0’ CP-treated splenocytes (Figure 7). Both intrinsic deficiency of TLR9 (TLR9^−/−^ mice) or blocking the effects of TLR9 (HCQ treatment) significantly reduced the production of IFNα and IFNβ. These results demonstrate that cfDNA and HMGB1 released from ischemically-injured cardiomyocytes activate the TLR9 – IFN-I pathway both inside the myocardium during ischemia and outside the myocardium during post-ischemic reperfusion. During ischemia, cfDNA/HMGB1 activate the TLR9 – IFN-I pathway, which leads to more ischemic injury and further cfDNA/HMGB1 release in addition to hypoxic injury, resulting in a vicious cycle that exacerbates ischemic myocardial injury. Deficiency of TLR9 or HCQ treatment before ischemia significantly reduced the production of IFNα and IFNβ (Figure 2B) and attenuated ischemic myocardial infarction (Figure 2A). Taken together, these results demonstrated that the TLR9 – IFN-I pathway inside myocardium was activated during ischemia and mediated ischemic myocardial infarction.

It has been demonstrated that post-ischemic reperfusion injury is induced by inflammatory responses [4,11,12,22–24,27] that are triggered by DAMPs released from ischemically-injured cardiomyocytes [11,12,29]. DAMPs thus are thought to exacerbate myocardial infarction during reperfusion [11]. We have demonstrated that two important DAMPs in particular, cfDNA and HMGB1, play a critical role in mediating inflammatory responses during reperfusion. Specifically, HMGB1 binds to RAGEs of inflammatory cells and facilitates intracellular migration of cfDNA [11,12]. Increased levels of cytosolic cfDNA then activate TLR9 and enhances production of IFN-Is [13]. The role of TLR9 in mediating ischemia/reperfusion injury has been explored in the liver [30] and heart [28,31]. In the current study, we found that TLR9^−/−^ mice had significantly smaller IS following 40’/60’ IRI compared to WT control mice; HCQ failed to further decrease the IS in TLR9^−/−^ mice (Figure 4A&B), suggesting that its primary infarct-reducing action is via the TLR9 pathway. The role of TLR9 was further defined by using the TLR9 antagonists ODN-2088 and HCQ. Administration of a TLR9 antagonist (ODN-2088 or high-dose HCQ) before occlusion of the LCA similarly attenuated myocardial IS after 40’/60’ IRI in WT mice (Figure 4C). Interestingly, ODN-2088 failed to attenuate IS if administered before reperfusion (data not shown). However, HCQ administered either before or after LCA occlusion similarly attenuated IS (Figure 5A and 4C). HCQ significantly decreased the plasma level of IFNα and IFNβ (Figure 5B), again supporting its inhibitory effects on the TLR9 – IFN-I pathway.

We further defined the role of TLR9 in mediating myocardial IRI with the use of a selective TLR9 agonist, ODN-1826. ODN-1826 and 40’/0’ WT CP significantly exacerbated IS in WT mice that underwent 20’/60’ IRI (Figure 6). Our results are consistent with a recent report demonstrating that ODN-1826 increases myocardial IRI [31]. WT 40’/0’ CP contained high levels of cfDNA and HMGB1 (Figure 3). We have demonstrated that cfDNA and HMGB1 in CP trigger the inflammatory response by activating the RAGE-TLR9 pathway [11,12]. Using 40’/0’ CP to treat isolated splenocytes, we found that CP stimulated splenocytes to secrete IFNα and IFNβ (Figure 7). These results further elucidate the role of the TLR9 – IFN-I pathway in mediating myocardial IRI.

There are multiple limitations to this study. Though HCQ was recently found to inhibit both TLR7 and TLR9, the role of TLR7 was not directly studied in this set of experiments. This might help to explain why ODN-2088 failed to attenuate IS when administered before reperfusion. However, the role of TLR7 in myocardial ischemia and IRI may be less significant than TLR9 given that IS in TLR9^−/−^ mice did not change with administration of HCQ. There are additional limitations with the clinical applicability of pre-ischemic treatments in particular. Treatment with HCQ before the onset of ischemia could likely protect the heart both during ischemia and during post-ischemic reperfusion. However, this is clinically not applicable unless patients are already taking HCQ regularly for another indication. Additionally, the dose required for attenuation of ischemia and IRI may be different than doses used in current practice. In the present study, high-dose HCQ tended to have the most significant impact on IS, which raises concerns about toxicity once translated clinically. There are numerous cardiovascular, renal, and metabolic effects that can be detrimental with high levels of HCQ [32]. Regarding the mode of administration, HCQ has previously been reported to be cardioprotective against myocardial IRI when administered orally for several days before the IRI [21]. However, all experiments in this study utilized intravenous HCQ. Thus, further research is required to determine the appropriate dosage of HCQ as well as the ideal mode of administration.

## 5. Conclusion

TLR9 – IFN-I-mediated inflammatory response contributes importantly to both ischemic and post-ischemic myocardial injury. HMGB1 and cfDNA released from ischemic myocardium activated the intra-myocardial TLR9 – IFN-I inflammatory pathway during ischemia and extra-myocardial TLR9 – IFN-I inflammatory pathway during reperfusion. Hydroxychloroquine reduces production of IFN-I and attenuates myocardial IRI, likely by inhibiting TLR9. The use of hydroxychloroquine during acute myocardial infarction to reduce infarct size provides a potential promising new use for a drug currently on the market. Future studies to further characterize its use in this field are warranted.

## List of Abbreviations:

MI: Myocardial Infarction
IRI: Ischemia/Reperfusion Injury
LV: Left Ventricle
DAMPs: Damage-Associated Molecular Patterns
PRRs: Pattern Recognition Receptors
TLR: Toll-Like Receptors
cfDNA: Cell-Free DNA
HMGB1: High Mobility Group Box-1
RAGE: Receptor for Advanced Glycation Endproducts
pDCs: Plasmacytoid Dendritic Cells
IFN-Is: Type-I Interferons
IFNα: Interferon Alpha
IFNβ: Interferon Beta
HCQ: Hydroxychloroquine
WT: Wild Type
ODN: Oligodeoxynucleotides
PBS: Phosphate Buffered Saline
CP: Cardiac Perfusate
LCA: Left Coronary Artery
IS: Infarct Size
RR: Risk Region
